# Antibodies from Rabbits Immunized with HIV-1 Clade B SOSIP Trimers Can Neutralize Multiple Clade B Viruses by Destabilizing the Envelope Glycoprotein

**DOI:** 10.1128/JVI.00094-21

**Published:** 2021-08-10

**Authors:** M. M. van Haaren, L. E. McCoy, J. L. Torres, W. Lee, C. A. Cottrell, J. L. Copps, P. van der Woude, A. Yasmeen, S. W. de Taeye, A. Torrents de la Peña, J. P. Moore, D. R. Burton, P. J. Klasse, A. B. Ward, R. W. Sanders, M. J. van Gils

**Affiliations:** a Department of Medical Microbiology, Amsterdam Infection & Immunity Institute, Amsterdam UMC, location AMC, University of Amsterdam, Amsterdam, The Netherlands; b Department of Immunology and Microbiology, The Scripps Research Institute, La Jolla, California, USA; c Division of Infection and Immunity, University College London, London, United Kingdom; d Department of Integrative Structural and Computational Biology, The Scripps Research Institute, La Jolla, California, USA; e Department of Microbiology and Immunology, Weill Medical College of Cornell University, New York, New York, USA; f International AIDS Vaccine Initiative–Neutralizing Antibody Center (IAVI-NAC), The Scripps Research Institute, La Jolla, California, USA; g Center for HIV/AIDS Vaccine Development (CHAVD), The Scripps Research Institute, La Jolla, California, USA; Emory University

**Keywords:** HIV-1, vaccine, monoclonal antibodies, AMC008 SOSIP, trimer destabilization, approach angle, human immunodeficiency virus

## Abstract

The high viral diversity of HIV-1 is a formidable hurdle for the development of an HIV-1 vaccine. Elicitation of broadly neutralizing antibodies (bNAbs) would offer a solution, but so far immunization strategies have failed to efficiently elicit bNAbs. To overcome these obstacles, it is important to understand the immune responses elicited by current HIV-1 envelope glycoprotein (Env) immunogens. To gain more insight, we characterized monoclonal antibodies (MAbs) isolated from rabbits immunized with Env SOSIP trimers based on the clade B isolate AMC008. Four rabbits that were immunized three times with AMC008 trimer developed robust autologous and sporadic low-titer heterologous neutralizing responses. Seventeen AMC008 trimer-reactive MAbs were isolated using antigen-specific single B-cell sorting. Four of these MAbs neutralized the autologous AMC008 virus and several other clade B viruses. When visualized by electron microscopy, the complex of the neutralizing MAbs with the AMC008 trimer showed binding to the gp41 subunit with unusual approach angles, and we observed that their neutralization ability depended on their capacity to induce Env trimer dissociation. Thus, AMC008 SOSIP trimer immunization induced clade B-neutralizing MAbs with unusual approach angles with neutralizing effects that involve trimer destabilization. Optimizing these responses might provide an avenue to the induction of trimer-dissociating bNAbs.

**IMPORTANCE** Roughly 32 million people have died as a consequence of HIV-1 infection since the start of the epidemic, and 1.7 million people still get infected with HIV-1 annually. Therefore, a vaccine to prevent HIV-1 infection is urgently needed. Current HIV-1 immunogens are not able to elicit the broad immune responses needed to provide protection against the large variation of HIV-1 strains circulating globally. A better understanding of the humoral immune responses elicited by immunization with state-of-the-art HIV-1 immunogens should facilitate the design of improved HIV-1 vaccine candidates. We identified antibodies with the ability to neutralize multiple HIV-1 viruses by destabilization of the envelope glycoprotein. Their weak but consistent cross-neutralization ability indicates the potential of this epitope to elicit broad responses. The trimer-destabilizing effect of the neutralizing MAbs, combined with detailed characterization of the neutralization epitope, can be used to shape the next generation of HIV-1 immunogens to elicit improved humoral responses after vaccination.

## INTRODUCTION

The ongoing HIV-1 epidemic, in spite of effective HIV-1 medication, highlights the need for an HIV-1 vaccine. To achieve this goal, knowledge of the immune responses elicited by state-of-the-art HIV-1 immunogens is important. Such knowledge will allow the further optimization and development of these immunogens. Many immunogens that are being explored as subunit vaccines are based on the HIV-1 envelope glycoprotein (Env) trimer (1–6). The Env trimer is the only viral protein expressed on the outside of the HIV-1 particle and therefore the only target for neutralizing antibodies (NAbs). Because circulating HIV-1 viruses have extremely diverse Env sequences, in order to provide protection, an HIV-1 vaccine needs to induce broadly neutralizing antibodies (bNAbs), i.e., NAbs that can cope with Env diversity (7). Extensive research has provided the field with soluble, stable, and native-like versions of Env, including SOSIP trimers (8). So far, SOSIP trimers have generally elicited strong autologous NAb responses, but only sporadic, inconsistent, and weak cross-NAb responses (9–12). It is imperative to study these antibody (Ab) responses to understand precisely which improvements are needed to consistently broaden the response. Iterative vaccine design based on monoclonal Abs (MAbs) isolated from vaccinated animals is a valuable way to overcome the limitations of the current HIV-1 immunogens (13, 14).

Previous studies characterizing MAbs and bulk serum of SOSIP Env trimer-immunized rabbits and macaques showed that the Ab responses frequently target strain-specific glycan holes (15–17). Indeed, the immunodominance of glycan holes was confirmed by redirection of vaccine-induced Ab responses toward *de novo* glycan holes when the original strain-specific glycan hole was filled (18). Env trimers from different virus isolates probably have their own specific immunodominant glycan holes, which would explain why Env trimer-immunized animals develop very limited neutralization breadth. Another immunodominant region after immunization is the unprotected base of the soluble Env trimer (17, 19, 20). This region of the Env trimer is, in its natural display, concealed by the viral membrane and in no need of heavy glycosylation to evade the immune system. However, on soluble Env trimers, the base forms a large glycan hole that is easily accessed by the immune system, and induces Abs that cannot recognize the full-length Env trimer, i.e., that are non-NAbs.

Many vaccine-induced NAbs target epitopes that overlap those of non-NAbs (11, 15, 16). Yet, it is unclear what exactly determines whether an Ab will have neutralizing ability. For some Ab families, binding kinetics might influence neutralization ability (21, 22). Indeed, it has been shown that some Ab families elicited during natural infection have kinetics of Ab binding that correlate with their neutralization ability. In particular, a high off-rate constant was associated with absent or less effective neutralization. The on-rate constant and overall affinity appeared to be of less influence on the ability to neutralize (21, 22).

SOSIP-induced NAbs have been discovered that target the same epitope as well-known bNAbs elicited after natural HIV-1 infection, but without displaying the same potency or breadth (17, 19, 23–25). Some studies have shown that the approach angle is relevant in this respect. For instance, for CD4-binding site (CD4bs)-directed Abs, the correct approach angle is essential for their ability to neutralize. The right approach angle allows bNAbs to reach the CD4bs while circumventing the dense Env glycan shield (23).

Several vaccines involving state-of-the-art Env trimer immunogens (1, 10) or specific Env epitope scaffolds (26, 27) have induced sporadic NAb responses against heterologous viruses in addition to autologous NAbs. Although such heterologous neutralization can be broad, spanning many clades, it is usually not very potent. Interestingly, two bNAbs have been isolated from a rabbit immunized with Env trimers on liposomes (1). This rabbit serum exhibited broad neutralization, and the isolated bNAbs recapitulated that activity. The development of breadth in this one rabbit was exceptional, as none of the other rabbits receiving the same immunogens developed this remarkable neutralization breadth, but the detailed characterization of such immune responses through the isolation of MAbs helps to understand why the development of neutralization breadth is rare and how it can be improved.

In this study, we isolated MAbs from four rabbits immunized with the clade B Env trimer immunogen AMC008 SOSIP. We identified an immunodominant area on the gp41 subunit of the AMC008 SOSIP trimer. Interestingly, these NAbs could cross-neutralize other clade B viruses. Negative-stain electron microscopy (NS-EM) revealed that these NAbs bound with an unusual approach angle that would be expected to be incompatible with binding to virus-associated Env trimers because of a clash with the viral membrane. Contrary to expectations and despite the unusual angle of approach, these MAbs were able to bind and neutralize, demonstrating remarkable flexibility of virus-associated Env trimers in their interaction with Abs (28). We further showed that the neutralization capacity of these NAbs depended on their ability to dissociate the Env trimer, similarly to what has been described previously for bNAbs isolated from both humans and rabbits (1, 29). The information gathered from this study helps to elucidate mechanistic aspects of virus neutralization and may help to tailor immunogens to elicit trimer-dissociating NAbs.

## RESULTS

### AMC008 SOSIP immunization induces NAbs and non-NAbs.

**FIG 1:**
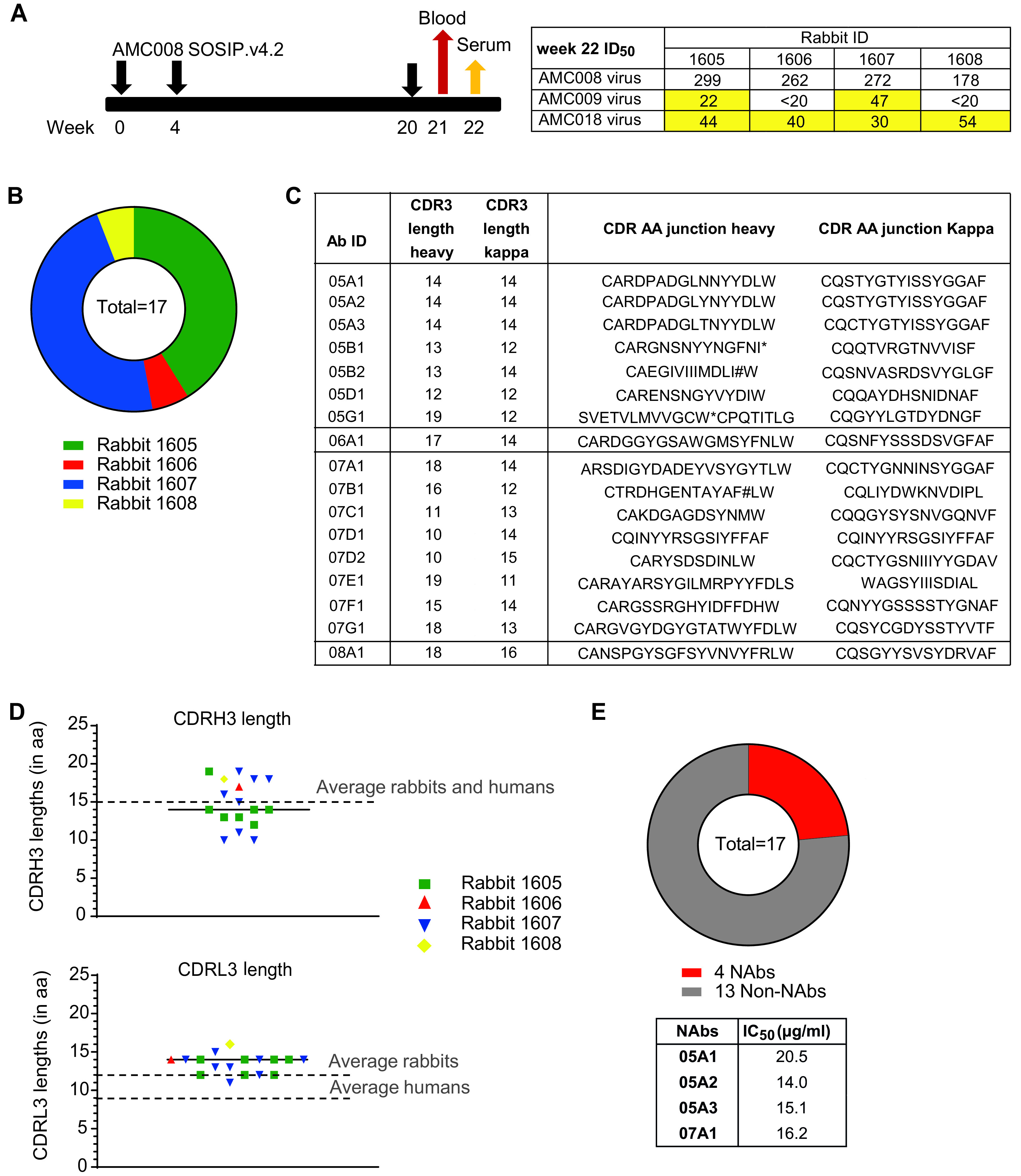
Characteristics of monoclonal antibodies (MAbs) isolated from AMC008 SOSIP trimer-immunized rabbits. (A) Immunization and sample collection scheme (left) and autologous and heterologous serum neutralization titers at week 22 (right) for four AMC008 SOSIP trimer-immunized rabbits. Serum neutralization 50% infective dose (ID_50_) is shown for each animal. (B) Absolute number of MAbs isolated per animal. (C) Individual CDR3 lengths and CDR amino acid junction sequence of heavy and light chains of all isolated MAbs. (D) Individual CDR3 amino acid lengths of the isolated MAbs. (E) Autologous neutralization ability of the isolated MAbs. Neutralization 50% inhibitory concentration (IC_50_) values are shown for each of the four neutralizing antibodies (NAbs) in the accompanying table.

In a previous study by our group, 15 rabbits (animal identifiers 1594 to 1608) were immunized with the clade B Env trimer AMC008 SOSIP (10). This immunogen was based on the viral sequence from an individual enrolled in the Amsterdam Cohort Studies (ACS) that showed broad serum neutralization (10). All AMC008 SOSIP trimer-immunized rabbits showed consistent autologous neutralization, as well as low cross-neutralization of the clade B viruses BaL, REJO, WITO, and SHIV162p3 (10). We further investigated serum neutralization of animals 1605 to 1608 and observed cross-neutralization at low titers by two and four rabbits, respectively, of the clade B viruses AMC009 and AMC018, isolated from different HIV-infected individuals (Fig. 1A). To better understand the cross-neutralization in these animals and its limitations, MAbs were isolated from these four AMC008 SOSIP trimer-immunized rabbits. Peripheral blood mononuclear cells (PBMCs) were obtained from these animals at week 21, 1 week after the third immunization (Fig. 1A). Single B cells expressing IgG, and with the ability to bind two distinctly labeled fluorescent AMC008 SOSIP trimers, were selected by fluorescence-activated cell sorting (FACS). On average, 5% of total live B cells were AMC008 SOSIP trimer reactive. B-cell receptor (BCR) sequences were subsequently amplified and cloned into expression vectors to generate MAbs that were tested for binding to autologous AMC008 SOSIP trimers by enzyme-limited immunosorbent assay (ELISA). A total of 17 MAbs bound the AMC008 SOSIP trimer. Rabbits 1605 and 1607 yielded 7 and 8 MAbs, respectively, while from the other two rabbits (1606 and 1608), only one MAb each was generated (Fig. 1B).

Sequence analysis of the heavy chain variable region showed the expected polyclonal immune response within each rabbit (Fig. 1C). From these sequences, we determined the length of the complementary determining region 3 of the heavy and the light chains (CDRH3 and CDRL3, respectively). The CDRH3 is important, as it interacts directly with the immunogen and is often elongated in human bNAbs to cope with the extensive glycan shield surrounding the Env trimer (30, 31). We also analyzed the CDRL3 because rabbit Abs, in contrast to human Abs, usually interact with the immunogen predominantly through the CDRs of their light chain (32). The average length of the CDRH3 of all AMC008 SOSIP trimer-reactive MAbs was 15 amino acids, which agrees with previous studies on CDRH3 lengths of BCR sequences in naive rabbits (32, 33) (Fig. 1D). However, the individual CDRH3 length varied greatly, ranging from 10 to 22 amino acids. The average CDRL3 length was increased with 2-amino-acid residues to an average of 14 residues in AMC008 SOSIP trimer-reactive MAbs from 12 residues in BCR sequences from naive rabbits (32, 33). The distribution of the individual CDRL3 lengths in the rabbits was more limited than that of the CDRH3 region, ranging from 11 to 16 amino acids (Fig. 1D).

We then tested the isolated MAbs for their ability to neutralize the autologous AMC008 virus (Fig. 1E). Four of the 17 MAbs were able to neutralize the AMC008 virus, although with relatively low potency (50% inhibitory concentration [IC_50_] values ranging from 16 to 19 μg/ml). Three of the identified four NAbs were isolated from rabbit 1605 and belonged to the same clonal family; these were designated 05A1, 05A2, and 05A3. The fourth NAb was isolated from rabbit 1607 and named 07A1. Alignment of heavy and light chain variable regions revealed 93% sequence similarity between the CDRL3 sequences of the 05A family (Fig. 1C). In addition, we found no evidence of gene conversion, a common feature for rabbit Abs, within this family of 3 MAbs. Interestingly, NAb 07A1 CDRL3 region was similar to that of 05A1-3 (79% sequence similarity). This shared CDRL3 was relatively long, with a length of 14 amino acids. The heavy chain CDR sequences of the 05A family and that of 07A1 did not show any similarities (Dig. 1C).

### AMC008 SOSIP-induced NAbs cross-neutralize some clade B viruses.

The cross-binding ability of the NAbs was tested against a panel of 12 heterologous SOSIP Env trimers (Table 1). This panel included four clade B SOSIP Env trimers (AMC009, AMC011, AMC016, and AMC018) based on virus sequences from individuals enrolled in the ACS (34). The Env sequences from these four clade B SOSIP trimers have >83% sequence identity with the AMC008 Env protein. Additional clade B SOSIP Env trimers derived from SHIV162p3 and REJO, showing 79% and 84% sequence identity, respectively, with AMC008, were also included in the panel, as neutralization of these viruses by the corresponding rabbit sera was observed previously (10). Furthermore, we included a selection of non-clade-B SOSIP trimers from a representative global panel, i.e., CNE55 (clade CRF01_AE), BJOX002000.03.2 (clade CRF07_BC), Ce1176_A3 (clade C), and 25710-2.43 (clade C) (35), as well as BG505 (clade A) (5) and, finally, the ConM SOSIP trimer, a consensus sequence protein based on the consensus sequences of each individual HIV-1 group M clade (36). All four NAbs displayed cross-reactivity with five out of the six clade B SOSIP Env trimers (Table 1). The NAb family 05A1-A3 also bound to the clade C SOSIP trimer Ce1176_A3, but there was no binding to other SOSIP trimers, suggesting that the target epitope is fairly conserved in clade B isolates but not across different clades.

**TABLE 1:**
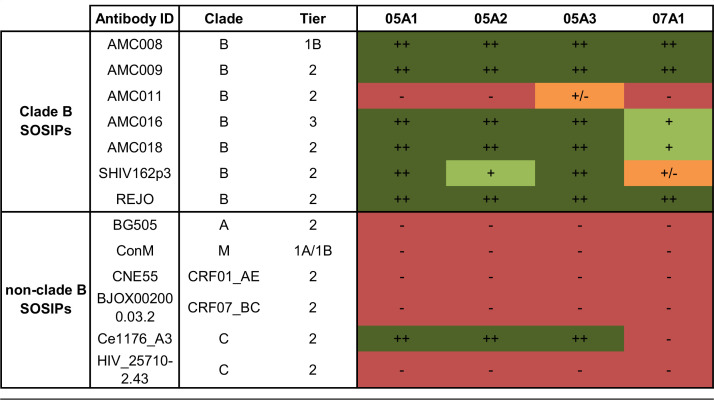
Heterologous binding ability of the isolated NAbs^*a*^

a Enzyme-limited immunosorbent assay (ELISA) cross-binding ability (50% effective concentration [EC_50_] in μg/ml) of the isolated AMC008 SOSIP trimer-reactive neutralizing antibodies (NAbs) to various clade B and non-clade-B SOSIP Env trimers. Env trimer binding ability is depicted with colors and symbols, where green colored cells (++ and +) indicate EC_50_ higher or equal to AMC008 binding titers, orange (+/−) indicates lower IC_50_ s and red (−) indicates no binding.

All four NAbs were then tested for neutralization breadth against a panel of 17 heterologous viruses consisting of a subpanel representing the global diversity of HIV-1 supplemented with a number of clade B viruses (35). Two of the viruses in this heterologous panel, the clade B tier 2 viruses SHIV162p3 and AMC009, were cross-neutralized by all four NAbs (Table 2). SHIV162p3 was neutralized relatively weakly, with IC_50_ values ranging from 5.6 to 19 μg/ml, i.e., similar to those against the autologous AMC008 virus. Heterologous AMC009 neutralization by the NAbs was much weaker, with IC_50_ values ranging from 58 to 177 μg/ml. The relative cross-neutralization of autologous AMC008 and heterologous SHIV162p3 and AMC009 is consistent with the cross-neutralization titers of the sera of rabbits 1605 and 1607 (10).

**TABLE 2:**
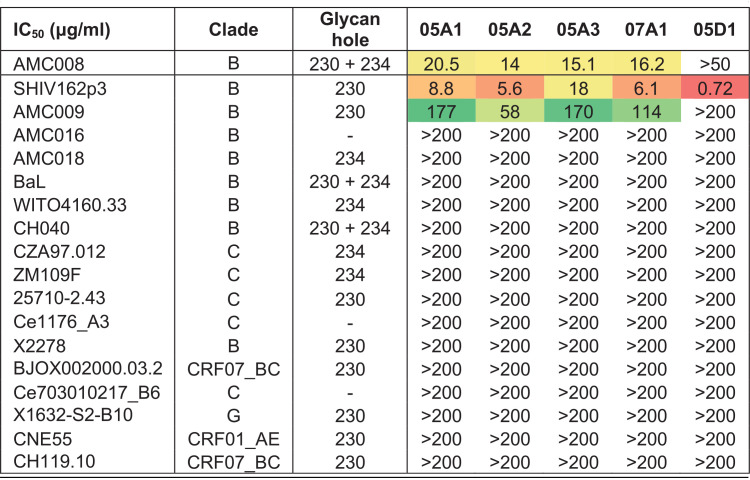
Neutralization ability (IC_50_ in μg/ml) of the autologous NAbs to neutralize a panel of 17 heterologous viruses.

### AMC008 NAbs target an epitope on the gp41 subunit.

**FIG 2:**
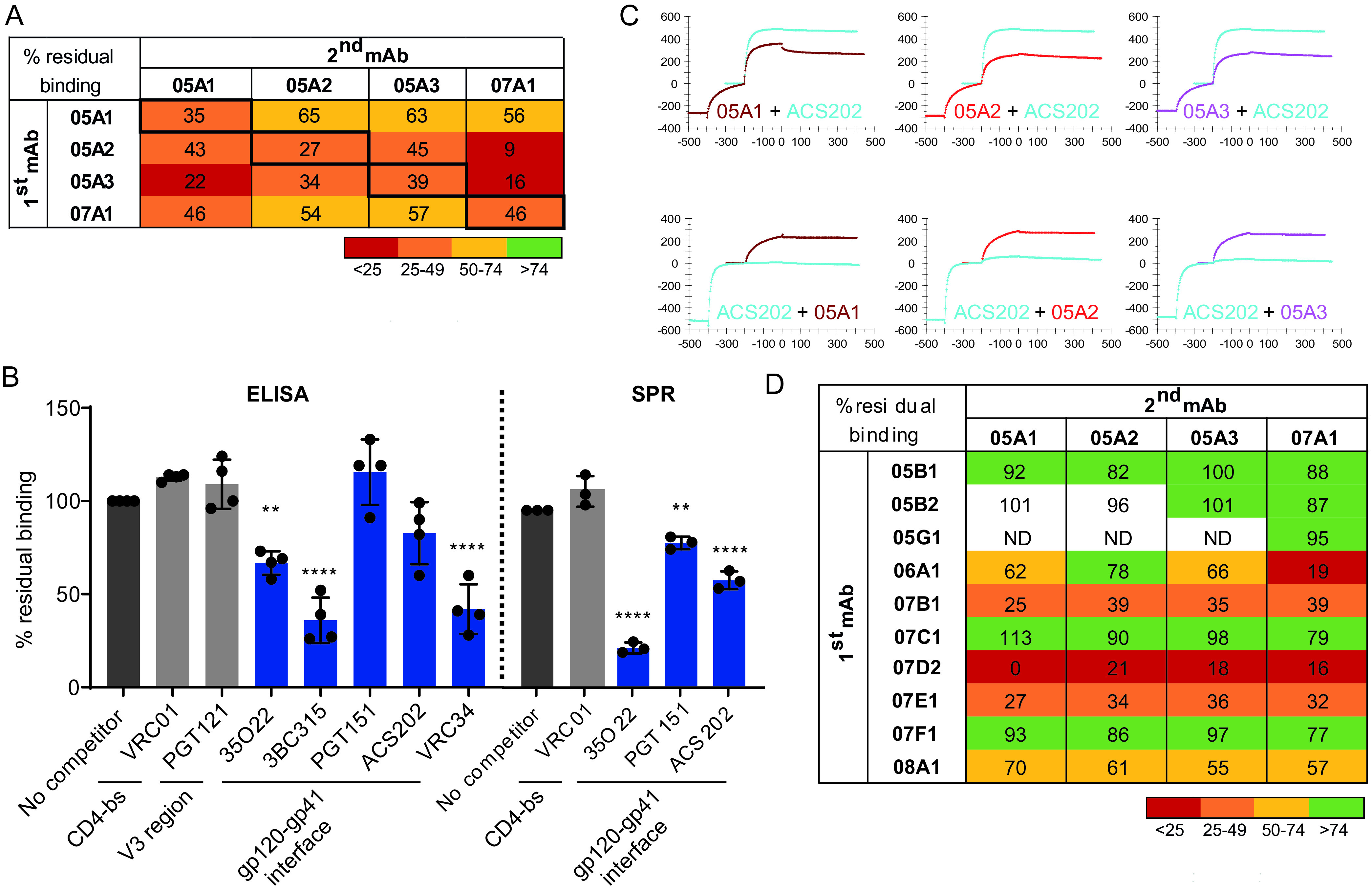
Competitive assays of NAbs with bNAbs and non-NAbs to specify an epitope. (A) Percent residual binding of NAbs to AMC008 SOSIP trimers in the presence of a competing NAb. The percent residual binding was calculated as follows: (shift in nm at 600 s of 2nd MAb binding × 100)/(shift in nm of 2nd MAb in the absence of 1st MAb binding). (B) Competition enzyme-limited immunosorbent assay (ELISA) and surface plasmon resonance (SPR) data of all NAbs with human broadly neutralizing antibodies (bNAbs). The percent residual binding was calculated as follows. For ELISA, (average optical density at 450 nm [OD_450_] of a triplo in the presence of the 2nd MAb × 100)/(average OD_450_ of a triplo in the absence of the 1st MAb binding); for SPR: [(response difference at 200 s for the second Ab)/(response difference at 200 s for the same, second Ab when injected as a single Ab in a separate cycle)] × 100 (%). Significant results are highlighted by asterisks (**, *P* < 0.005; ***, *P* < 0.0001). SPR was only performed for the 05A family of NAbs. (C) SPR binding curves of AMC008 SOSIP trimer binding competition between NAbs and the bNAb ACS2020 that shows the influence of assay directionality. Ab binding is recorded in real time; the *x* axis indicates the time in seconds. The *y* axis shows the response (response units [RU]), proportional to the mass bound. Dissociation starts at 0 s. The top three graphs show binding of ACS202 in the presence of competitors 05A1 to 05A3 (dark red, red, and purple lines), compared to ACS202 binding in the absence of these NAbs (blue lines). The lower three graphs show a reverse assay setup, showing binding ability of NAbs 05A1 to 05A3 in the presence of competitor ACS202 (blue lines) and in the absence of ACS202 (dark red, red, and purple lines). (D) Competition results of the four rabbit NAbs with the isolated non-NAbs, measured by BLI. Percent residual binding was calculated as stated for Fig. 2A.

Because the four NAbs had nearly identical CDRL3 sequences and three of them were clonal family members, we hypothesized that they might target a shared epitope. To test this, we performed competition assays between these four NAbs using biolayer interferometry (BLI). All NAbs showed strong and reciprocal competition with each other, suggesting that their epitopes overlap (Fig. 2A). Differences in percentage of residual binding were observed depending on the directionality of the assay. For instance, we observed 54% residual binding of 05A2 after preincubation with competitor 07A1 MAb. However, only 9% residual binding of 07A1 was measured when 05A2 was used as the competitor. These differences could be due to differences in affinity of the NAbs. All MAbs showed self-competition (46 to 27% residual binding) (Fig. 2A).

We then performed competition assays with known human bNAbs using ELISA and surface plasmon resonance (SPR) to specify the epitope targeted by these four NAbs. We tested competition with bNAbs VRC01, PGT121, 35O22, 3BC315, PGT151, and ACS202. These bNAbs target diverse regions on the Env trimer enabling us to define a potential binding area of NAbs 05A1-A3 and 07A1. All four NAbs competed significantly in ELISA with bNAbs 35O22, 3BC315, and VRC34, which all target gp41. Competition of the four NAbs 05A1, 05A2, 05A3, and 07A1 with 35O22 was weakest, with residual binding being 58%, 69%, 73%, and 67%, respectively (Fig. 2B). The four NAbs competed more efficiently with VRC34 and 3BC315, with residual binding between 25% and 60% (Fig. 2B). Weak but not statistically significant competition was observed with gp41–gp120-targeting bNAb ACS202 and no statistically significant competition was detected with gp41–gp120-targeting bNAb PGT151, or bNAbs VRC01, or PGT121, which bind to the CD4bs and the V3-N332 glycan epitope, respectively.

SPR analyses strengthened the evidence for competition of the 05A family with gp41–gp120-targeting bNAbs 35O22 (∼25% residual binding) and ACS202 (∼60% residual binding), and also revealed weak competition with PGT151 (∼80% residual binding) (Fig. 2B). The competition with ACS202 was enhanced when the SPR assay set-up was reversed. In this reversed set-up, where ACS202 was allowed to bind first and 05A1-3 second, the residual binding dropped to ∼10% (Fig. 2C). No significant competition of the rabbit NAbs with the bNAbs VRC01 and PGT121 was observed in the SPR assays in either set-up (Fig. 2B). These competition results suggest that the AMC008 SOSIP induced NAbs that most likely target an epitope at or near the gp41-gp120 interface area.

We also tested competition of the AMC008 SOSIP trimer-induced NAbs with the non-NAbs elicited against the same immunogen, using BLI. The binding of the four NAbs to the AMC008 SOSIP trimers was completely or partially abrogated when any of the five non-NAbs 06A1, 07B1, 07D2, 07E1, or 08A1 was present. These competition results suggest that these five non-NAbs have overlapping epitopes with the NAbs (Fig. 2D). The remaining five non-NAbs did not compete with the NAbs, suggesting these target different epitopes on the Env trimer.

**FIG 3:**
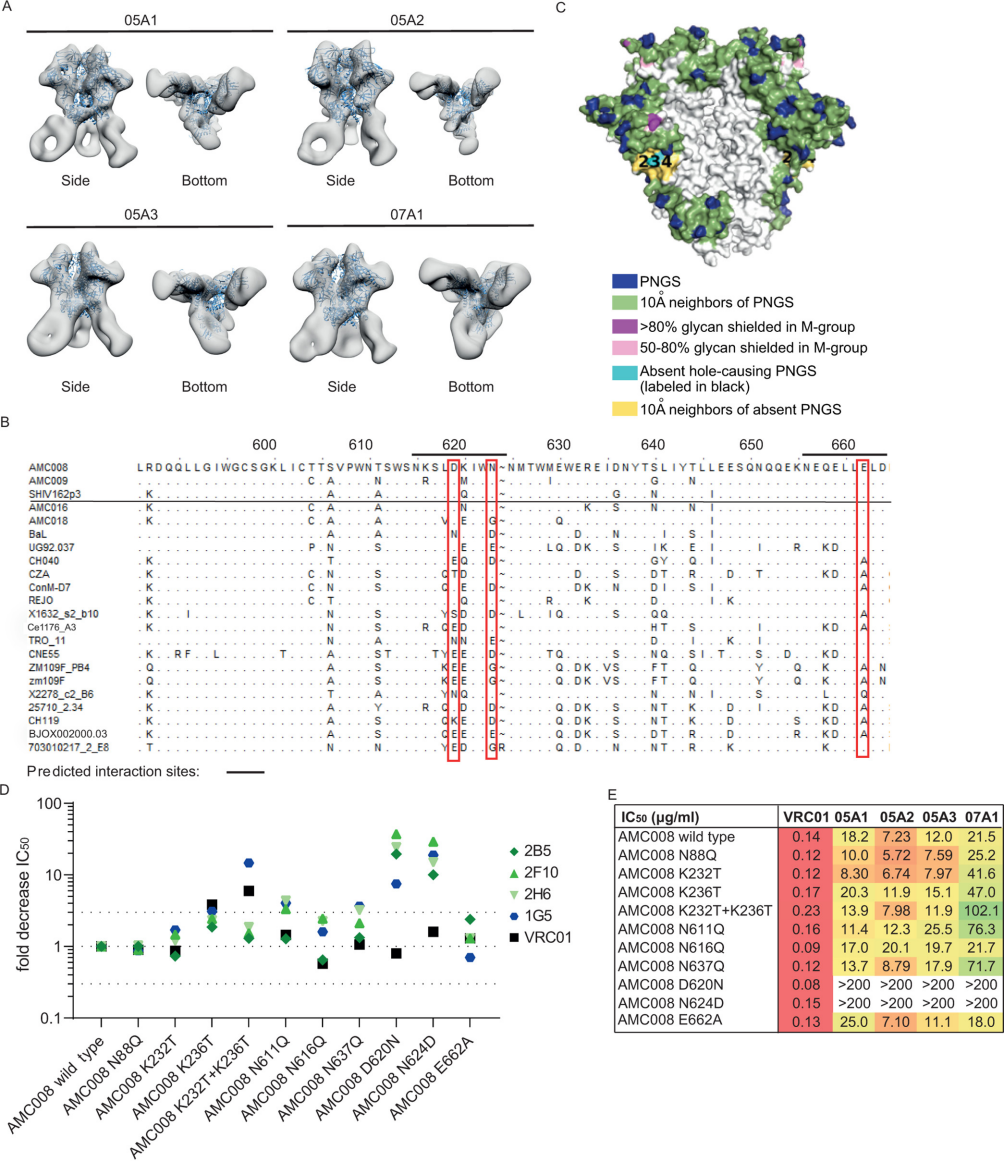
Epitope mapping of NAbs to the gp41 subunit of Env. (A) Negative-stain electron microscopy (NS-EM) 3D reconstructions of the NAbs Fab fragments in complex with the AMC008 SOSIP trimer. The AMC008 SOSIP trimer structure is modeled as the ribboned density. The NAbs are shown as white densities. Side and bottom views are depicted. (B) Alignment of viral sequences tested for neutralization and binding by 05A1 to 05A3 and 07A1. AMC008, AMC009, and SHIV162p3 could be neutralized by the NAbs, whereas the viruses below the line were not neutralized by the NAbs. HXB2 amino acid numbering is indicated on top, and lines indicate the NAb binding sites predicted by NS-EM imaging. Red boxes show which amino acid residues were mutated in the predicted binding sites to specify the epitope, namely, D620N, N624D, and E662A. (C) Model of the glycan shield present on the AMC008 SOSIP trimer with a strain-specific glycan hole indicated in cyan and yellow due to the absence of PNGS 230 and 234. Image was created with the glycan shield mapping tool on the Los Alamos database (57). (D) Neutralization ability of NAbs to multiple viral variants. Fold decrease in IC_50_ values is plotted for each of the NAbs and the VRC01 control, each represented by different colors. The dotted lines indicate a 3-fold threshold compared to AMC008 wild-type neutralization IC_50_ values. (E) Neutralization ability of the NAbs for various AMC008 mutants. IC_50_ values are indicated in μg/ml. The bNAb VRC01 is taken along as a positive control.

Negative-stain electron microscopy (NS-EM) was performed to further delineate the NAb epitope and to confirm its location at or near the gp41-gp120 interface. Binding of Fab fragments from the four identified NAbs to the AMC008 SOSIP trimer was visualized through three-dimensional (3D) reconstructions. The NAb Fabs interacted with the gp41 subunit of the AMC008 SOSIP trimer, possibly interacting with residues S528 to A532, N616 to N625, and Q658 to D644 in the HR2 region (Fig. 3A). Additional NS-EM analysis carried out with 05A3 revealed that it bound with a predominant stoichiometry of two Fabs to one Env trimer, although full occupancy of three Fabs binding to one trimer could be detected in a minority of cases. Interestingly, the NAbs interacted with the Env trimer with unusual approach angles that would *a priori* be expected to lead to a clash of the NAbs with the viral membrane.

### AMC008 NAbs target residues 620 and 624 in HR2.

**FIG 4:**
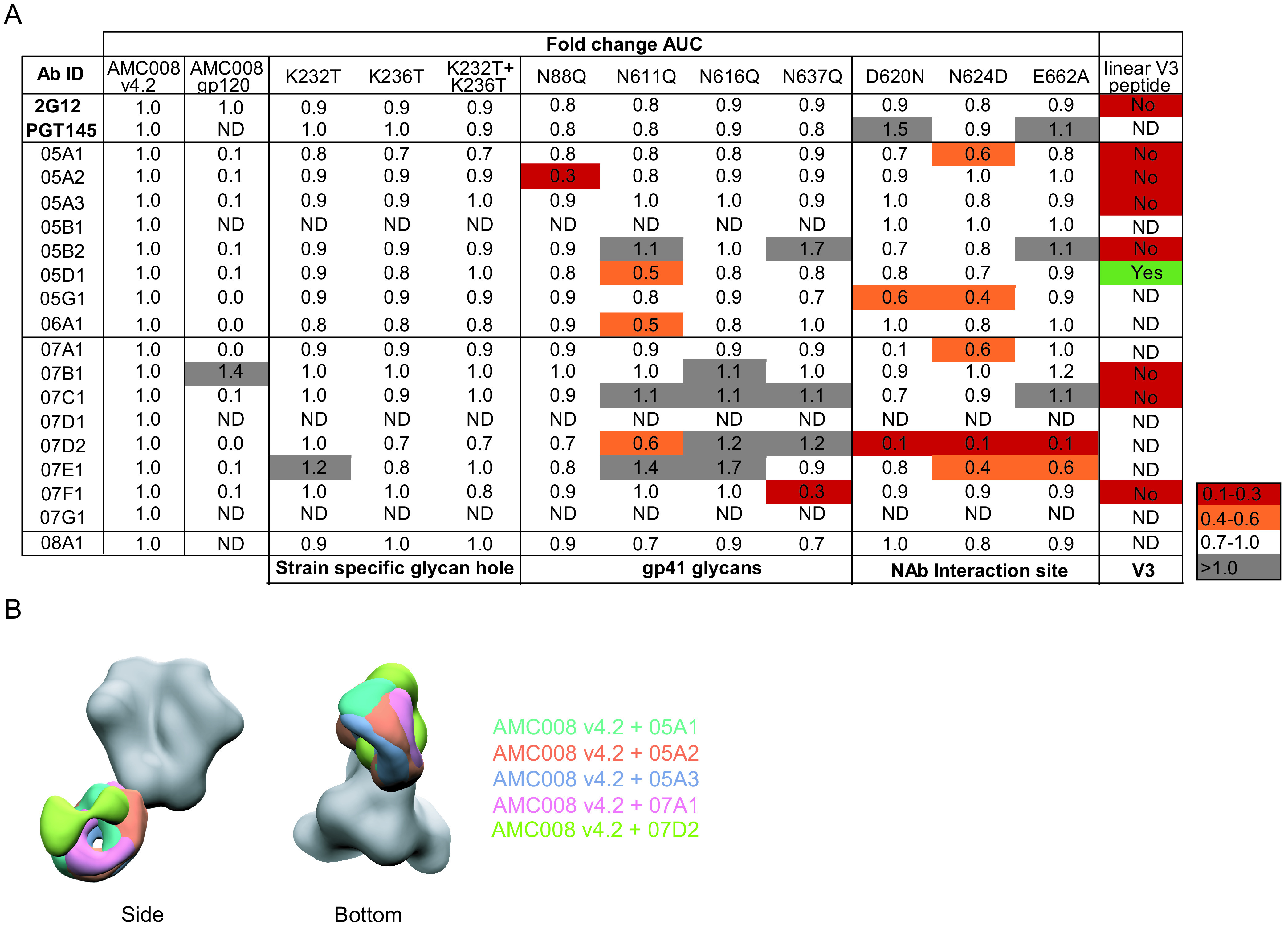
Epitope mapping of the nonneutralizing antibodies. (A) Binding ability of all isolated MAbs to various AMC008 mutants and linear V3 peptide. Fold change in area under the curve is displayed relative to AMC008 binding. Binding to the V3 peptide is indicated as “yes” or “no.” (B) NAbs 05A1 to 05A3 (purple and pink) and 07D2 (gray) were complexed with the AMC008 SOSIP Env and analyzed by NS-EM with the AMC008 SOSIP trimer to show differences in their binding angle.

The NS-EM analysis enabled us to make informed changes to identify amino acid residues important for binding and neutralization. Within predicted interaction regions pinpointed by the NS-EM analysis, we searched for differences between the sequences of Env trimers that the NAbs were able to cross-bind and/or neutralize and those of trimers that they could not bind or neutralize (Fig. 3B). Divergent amino acids were mutated in the context of the AMC008 SOSIP trimer and the corresponding pseudovirus to the most prevalent amino acid in the nonbinding sequences, resulting in three variants with single amino acid substitutions, i.e., D620N, N624D, and E662A. In addition, considering that Env glycans might influence NAb binding, we modified the glycan shield near or on gp41 of the AMC008 trimer. Accordingly, knockout (KO) mutants of the potential *N*-linked glycosylation sites (PNGS) at positions 88, 611, 616, and 637 were created. Finally, since previous studies showed that SOSIP-induced NAbs often target strain-specific holes in the glycan shield (15–17, 25), we knocked in the PNGS at positions 230 and 234 of the AMC008 sequence, as the absence of PNGS at these positions in the natural AMC008 sequence is expected to create a strain-specific glycan hole (Fig. 3C). Substitution of amino acids 620 and 624 abolished neutralization ability of all four NAbs, but not of the control Ab VRC01, consistent with the NS-EM data (Fig. 3D and E). Furthermore, neutralization by 07A1 was also affected by simultaneously introducing the N230 and N234 PNGS, although these substitutions did not completely abrogate activity, as neutralization of the virus still occurred at higher Ab concentrations. This suggests that glycans at N230 and N234 restrict access to the 07A1 epitope. Three out of four NAbs were also affected by removing the N611 and N637 PNGS; these glycans might therefore contribute to (the presentation of) the NAb epitope. The K232T and K236T single-knock-in (KI) mutations did not affect neutralization for any of the four NAbs, nor did the N88 and N616 KO mutations or the E662A amino acid substitution. When residues 230, 234, 611, 620, 624, or 637 were mutated in the AMC008 SOSIP trimer context, it did not detectably affect binding of NAbs 05A1 to 05A3 in ELISA, while the effect on NAb 07A1 was only observed when residues 620 and 624 were changed (Fig. 4A). Differential effects of single mutations in neutralization versus binding assays have been observed in other cases (37, 38), and these probably relate to affinity and Env protein conformation and stability.

We were also able to define the epitopes for the majority of the non-NAbs. MAbs 07E1 and 07D2 also showed dependence on the 620, 624, and/or 662 amino acids in ELISA (Fig. 4A), confirming their epitope overlap with the NAbs, as suggested by the competition experiments. NS-EM further confirmed this epitope overlap for non-NAb 07D2 (Fig. 4B). Other non-NAbs bound a variety of epitopes on the gp41 subunit of the Env trimer, such as the area around the N637 glycan (07F1) (Fig. 4A) and an epitope at the base of the Env trimer (07B1). The non-NAb 05D1 was able to bind a linear V3 peptide in ELISA (Fig. 4A).

### NAbs and non-NAbs targeting the 620/624 site cannot be differentiated based on affinity for soluble trimers.

**FIG 5:**
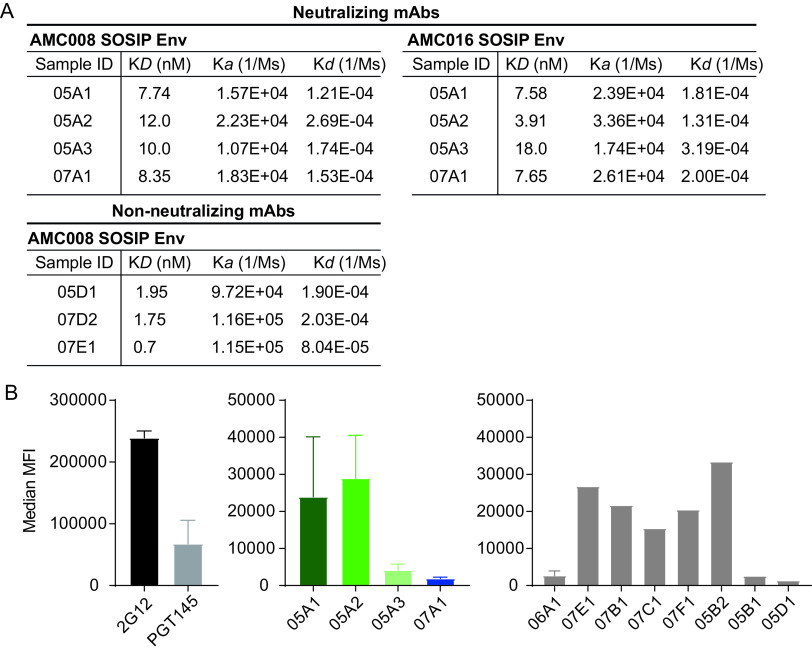
Analysis of neutralizing and nonneutralizing antibody characteristics that might influence neutralization. (A) Binding kinetics of NAbs and non-NAbs to the autologous AMC008 SOSIP and heterologous AMC016 SOSIP Env trimers. *K_D_*, on-rate (*K_a_*), and off-rate (*K_d_*) constants are shown. NAbs were tested for binding to AMC008 and AMC016 SOSIP Env trimers. Non-NAbs were only tested for binding to the autologous AMC008 SOSIP trimer. Results were fitted to a 1:1 kinetics model for analysis. (B) Binding to full-length surface-expressed AMC008 SOSIP gp160 Env by MAbs. Maximum mean fluorescent intensity (MFI) is plotted for each MAb tested. (Left) Binding and expression controls (2G12 and PGT145, respectively). (Middle) Binding of the NAbs to full-length AMC008 gp160 Env trimers. (Right) Binding of a selection of non-NAbs to full-length AMC008 gp160 Env trimers.

We were intrigued by the identification of NAbs and non-NAbs that targeted overlapping epitopes. A first hypothesis would be that binding affinity might explain the differences in neutralization ability between NAbs and non-NAbs. To test this, we subjected a subset of AMC008 SOSIP-induced non-NAbs and all four NAbs to kinetic binding experiments with AMC008 SOSIP Env trimers. No significant differences were observed in affinity between the NAbs and the non-NAbs (*P* = 0.057 for *K_D_* [equilibrium dissociation constant], *P* = 0.057 for *K_a_* [association constant], and *P* > 0.999 for *K_d_* [dissociation constant]; Mann-Whitney U test) (Fig. 5A).

We also noted that all four NAbs were able to cross-bind certain SOSIP Env trimers, but were unable to neutralize their corresponding virus. We asked whether this observation could be explained by differences in affinity as well. We tested all four of the NAbs in kinetic binding experiments using AMC008 SOSIP and AMC016 SOSIP Env trimers. The latter was selected in addition to the autologous trimer because the AMC016 virus was not neutralized by our NAbs, while they were able to bind to the corresponding AMC016 SOSIP trimer (Table 2). AMC016 SOSIP trimers contain the amino acids essential for neutralization (D620 and N624), but also the N230 and N234 PNGS that are absent from the AMC008 Env sequence. Kinetic analysis did not reveal significant differences in NAb binding kinetics between the AMC008 SOSIP and AMC016 Env trimers (*P* = 0.343 for *K_D_*, *P* = 0.114 for *K_a_*, and *P* = 0.486 for *K_d_*; Mann-Whitney U test) (Fig. 5A), suggesting that binding kinetics are not the main cause for the (in)ability of these NAbs to neutralize these viruses.

### NAbs and non-NAbs targeting the 620/624 site cannot be differentiated based on binding to membrane-associated Env trimers.

The next hypothesis posits that the discrepancy between binding and neutralization ability of the autologous AMC008 SOSIP Env trimer-reactive MAbs might be due to an inability of the non-NAbs to bind full-length surface-displayed Env trimers, possibly related to the observed unusual approach angle. To test this, we transfected HEK293 cells with AMC008 SOSIP gp160 Env constructs (39), which resulted in surface-expressed AMC008 SOSIP trimers that were subsequently analyzed by FACS to detect MAb binding (Fig. 5B). In contrast to what we expected, we found that NAbs and non-NAbs bound similarly to full-length cell surface-displayed AMC008 SOSIP gp160 trimers. Surprisingly, we found that the NAb 05A3 bound weakly, and NAb 07A1 was unable to bind cell surface-displayed SOSIP gp160 trimers, which is somewhat inconsistent with their ability to neutralize.

### Neutralization activity of MAbs against the 620/624 epitope is determined by their ability to disrupt the trimer.

**FIG 6:**
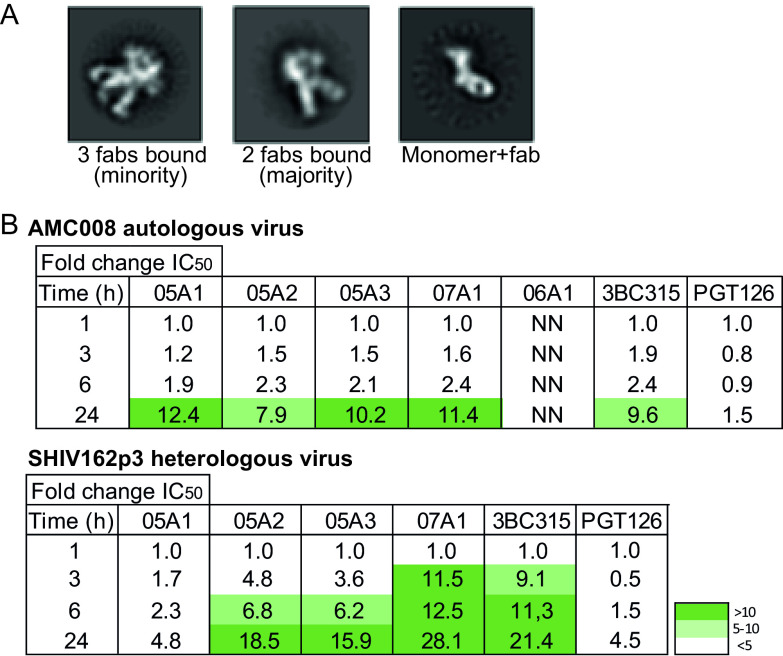
NAbs destabilize the trimer as a mechanism of neutralization. (A) Negative-stain electron microscopy images of AMC008 SOSIP trimers incubated overnight with the MAb 05A3. The majority of the images displayed MAb binding with a stoichiometry of 2 MAbs per trimer (middle). MAbs binding the monomeric form of the trimer were also observed (right). (B) Preincubation neutralization experiment to determine the *in vivo* destabilization ability of the isolated Abs. A neutralization assay was performed in which virus and Ab mix were incubated for different time periods, up to 24 h. The fold change in IC_50_ is shown for each of the Abs. The autologous AMC008 virus and heterologous SHIV162p3 viruses were tested. 3BC315 and PGT126 were tested as positive-control and negative-control Abs, respectively. Increased neutralization potency after the 24-h incubation step is seen for most of the NAbs and for 3BC315, but not for 06A1 and PGT126. NN, nonneutralizing.

A third hypothesis argues that the strength of neutralization is strongly influenced by the capability to induce trimer dissociation. Indeed, gp41 targeting human bNAbs 3BC315 and 3BC176 neutralize by inducing dissociation of the Env trimer, and so does rabbit bNAb 1C2 derived from an immunization study (1, 29). We noticed that the 05A3 Fab fragments caused the trimer to dissociate into monomers during sample preparation for NS-EM (Fig. 6A). Dissociation of the AMC008 SOSIP Env trimer did not occur spontaneously when the trimer was left overnight at room temperature in the absence of 05A1 or 05A3, suggesting that the Fab was responsible for the observed trimer dissociation.

To corroborate these findings, we performed a neutralization assay with prolonged preincubation times (29). This assay determines the neutralization ability of an Ab after preincubation over a 24-h time period at 37°C to measure irreversible trimer destabilization. An increase of neutralization with longer incubation times is indicative of trimer destabilization, as more trimers are destabilized by the Ab over time (1, 29). The bNAbs PGT126 and 3BC315 were tested in this assay as negative and positive controls, respectively, and showed an increase in neutralization potency of 1.5-fold and 10-fold, respectively, after 24 versus 1 h of incubation. The potency of our NAbs against the autologous AMC008 virus increased by ∼10-fold after 24 h of incubation (Fig. 6B), indicative of trimer dissociation and similar to the effect of 3BC315. The non-NAb 6A1, targeting a similar epitope as our NAbs, remained unable to neutralize the AMC008 virus even after 24 h of incubation. The results were even more pronounced with the heterologous SHIV162p3 virus, showing increases in potency of up to nearly 30-fold after 24 h. Interestingly, NAb 05A1 did show an increase in potency against the AMC008 virus over time, but no significant increase against the heterologous SHIV162p3 virus. Nevertheless, these data indicate that the four NAbs dissociate Env trimers of both autologous and heterologous viruses and that this largely contributes to their neutralization capacity.

## DISCUSSION

To overcome viral diversity, an HIV-1 vaccine should induce bNAbs. However, the currently used immunogens have been unable to consistently elicit such responses. In this study, we tried to better understand the humoral immune responses after HIV-1 SOSIP Env trimer immunization in rabbits. We set out to determine which epitopes on the clade B AMC008 SOSIP Env trimer were targeted and whether the observed specificities could explain the low-titer heterologous neutralization that was observed after immunization with this SOSIP trimer. We isolated and characterized 17 MAbs from four AMC008 SOSIP Env trimer-immunized rabbits. The vast majority of the isolated MAbs targeted a similar area in the HR2 region. One Ab family (05A) and the MAb 07A1 were found to neutralize the autologous and two heterologous viruses through destabilization of the Env trimer, a mechanism of neutralization also used by NAbs induced by natural infection or vaccination (1, 29). Additionally, we identified important contact residues for these NAbs. Such knowledge may help to shape next-generation SOSIP Env trimer immunogens to induce trimer-destabilizing NAbs.

Even though rabbits are a widely used animal model in the early stages of HIV-1 vaccine testing, we realize there are caveats to using this model. In contrast to humans, rabbits interact with the antigen predominantly through their light chain (32) and use one heavy V-gene in the initial Ab recombination process; therefore, diversity in the heavy chain is much more restricted compared to that in their light chains. Additionally, gene conversion and somatic hypermutation (SHM) are the major drivers of Ab diversity in rabbits; the former is a process that rarely takes place in humans (32, 40). Nonetheless, numerous isolated rabbit Abs show that similar epitopes on the Env trimer are targeted compared to those seen in macaques and humans after natural infection (15).

Although the NAbs that we describe do not have the desired breadth for a protective HIV-1 vaccine, they are rare examples of MAbs with consistent, albeit weak, cross-neutralizing ability, in particular after only three immunizations with only one immunogen. Other experiments have yielded only autologous neutralizing MAbs, with the notable exception of the isolation of two bNAbs from one Env-immunized rabbit (1, 16, 19, 25). Valuable information from these previous studies led to the development of new immunogens, such as Env trimers displayed on nanoparticles, germ line trimers, and specific epitope scaffolds that were expected to induce more broad and consistent neutralization (2, 27, 41, 42). Nonetheless, neutralization breadth in rabbits after HIV-1 immunization remains sporadic and is usually of low titer (9–12). The isolation of heterologous NAbs from multiple rabbits using the same neutralization mechanism and targeting similar epitopes highlights the possibility for immune focusing toward this epitope cluster. The identification of bNAbs isolated from both humans and rabbits that target similar epitopes, with the shared capacity to disrupt trimers, marks this epitope as a very interesting target to be further exploited (1, 29).

 bNAb 1C2, which exhibited an extraordinary broad NAb response (∼85%) was isolated from an Env-immunized rabbit (1). The mechanism of 1C2 neutralization involved destabilization of the Env trimer, similar to the effects of bNAbs 3BC315 and 3BC176 (29) and NAb family 05A and 07A1 described here. It was proposed that these bNAbs destabilize the trimer by disruption of the stabilizing tryptophan clasp formed by amino acids W623, W628, and W631 (1, 43). The CDRH3 loops of both rabbit bNAb 1C2 and human bNAb 3BC315 are close to residue W623 of this clasp. Because the NAbs we describe here have contacts with residues 620 and 624, neighboring this tryptophan clasp, it is not unlikely that a similar disruption mechanism is utilized by these rabbit NAbs. The description of multiple (b)NAbs directed to this epitope, with the same potent mechanism of action, indicate this epitope as a potential vaccine target. Nonetheless, the isolation of bNAbs, NAbs, and non-NAbs directed to this epitope shows that adaptations to the immunogen are necessary to amplify this desirable response and guide the responses to neutralization potency and breadth. One strategy involves glycan modifications to enhance accessibility of the tryptophan clasp. Another complementary strategy could involve immune focusing on conserved amino acids within this region, as opposed to ones that are more variable, such as residues 620 and 624.

The AMC008 SOSIP Env trimer lacks the N230 and N234 PNGS, generating a strain-specific glycan hole. The absence of these glycans might dictate the angle of approach for the NAbs identified in this study and thereby restrict the breadth of the response. Filling the 230/234 glycan hole negatively impacted neutralization by the NAb 07A1. However, this was not the case for the NAbs isolated from animal 1605. One explanation might be that the footprint of the 07A1 epitope is larger and/or in closer proximity to the 230/234 glycan hole. The ability of these NAbs to neutralize viruses containing the 230 and 234 PNGS, such as the SHIV162p3 virus, is encouraging, as the 234 PNGS is widely conserved.

The NS-EM revealed an improbable approach angle for the NAbs that should result in a clash with the viral membrane. It is possible that the angles by which the NAbs approach soluble Env trimers and virus-associated Env might differ, in particular at the trimer stem. For instance, the membrane-proximal external region (MPER) bNAb 10E8 Fab approaches soluble SOSIP trimers with an angle similar to that of 05A1 to 05A3 and 07A1, but this angle changes to facilitate the presence of the membrane when 10E8 binds to membrane-anchored Env (44). Moreover, viral membrane-associated envelope proteins can have remarkable flexibility. This flexibility was visualized for the severe acute respiratory syndrome coronavirus 2 (SARS-CoV-2) spike protein, which is able to tilt by up to ∼60° relative to a position perpendicular to the membrane (45). Also, HIV-1 Env trimers on nanodiscs displayed the ability to tilt by ∼20° when bound to gp41-directed bNAbs (28).

Previous studies suggested that a lower rate of dissociation was associated with stronger neutralization (21, 22). However, this did not apply to the gp41-directed MAbs described here. One difference is that these MAbs originated from different clonal families, whereas the previous studies analyzed members from the same family. Furthermore, we determined binding kinetics using stabilized soluble Env SOSIP trimers. The possibility cannot be excluded that Env stabilization in the SOSIP construct and the observed destabilization of the Env trimer by the Nabs might have affected the affinities we measured. Nevertheless, it is likely that a low dissociation rate and the ability to induce trimer dissociation are distinct properties that affect both neutralization potency and efficacy (46).

In summary, characterizing humoral immune responses at the MAb level after immunization can inform future immunization studies and strategies. We found that gp41-directed NAbs with an unusual approach angle that was predicted to clash with the viral membrane were able to weakly neutralize autologous and heterologous clade B viruses by inducing Env trimer destabilization, reminiscent of bNAbs that induce trimer dissociation by disrupting the tryptophan clasp in gp41. This knowledge highlights that destabilization of the trimer might be a more important neutralization mechanism than previously appreciated. Inducing trimer-destabilizing NAbs that target the tryptophan clasp should be considered in future immunization strategies.

## MATERIALS AND METHODS

### Isolation of rabbit peripheral blood mononuclear cells.

Blood of immunized rabbits was used for peripheral blood mononuclear cell (PBMC) isolation through Ficoll separation. In short, blood was diluted 1:1 with phosphate-buffered saline (PBS), loaded on a Ficoll layer, and centrifuged for 30 min at room temperature at 400 × *g* with an acceleration speed of 7 and a deceleration speed of 0. PBMCs were isolated, washed with PBS, and subsequently resuspended in 1 to 2 ml of ammonium-chloride-potassium (ACK) buffer (Thermo Fisher Scientific) to remove red blood cells. PBMCs were counted, suspended in fetal calf serum with 10% dimethyl sulfoxide (DMSO), and directly frozen at −150°C.

### Env protein design, production, and purification.

All SOSIP trimers contained the previously described SOSIP mutations (8) and were further stabilized with additional mutations, yielding SOSIP.v4.2 (E66K + A316W) trimers (10). ConM and SHIVp3 SOSIP trimers contained additional mutations (SOSIP.v9), described elsewhere (47–49). All SOSIP constructs were cloned into a pPPI4 expression vector (50). Mutant variants were generated by the use of the Q5 site-directed mutagenesis kit (New England Biolabs) with specifically designed primer sets and adapted annealing temperatures for the mutations N88Q, K232T, K236T, K232T+K236T, N611Q, N616Q, N637Q, D602N, N624D, and E662A. D7324 tags were incorporated directly C-terminal of residue 664 in each of the plasmids (8). In addition, we replaced the D7324 tag C-terminal of residue 664 with an Avi tag in the AMC008 SOSIP.v.4.2 construct for biotinylation (16). For BLI and SPR experiments an His tag was incorporated into AMC008 SOSIP.v4.2 and the AMC008 SOSIP.v4.2 D620N, N624D, and E662A mutant plasmids, replacing the D7324 tag (2).

All SOSIP trimer variants were produced as described before (6, 8, 10, 51, 52). In short, SOSIP trimers were transiently expressed together with furin from a separate expression plasmid (ratio, 4:1) in HEK293F cells (catalog no. R79009; Invitrogen). The SOSIP trimers were harvested by spinning for 20 min at 4,000 × *g*. The supernatant was 0.22 μM Steritop vacuum filtered before purification by gravity-driven chromatography on a PGT145 antibody-conjugated Sepharose column. Env proteins were eluted with 3 M Mg_2_Cl_2_ (pH 7.8), directly into neutralization buffer (20 mM Tris-HCl [pH 8.0] and 75 mM NaCl). After purification, Env proteins were concentrated with Vivaspin 100-kDa filters (GE Healthcare), to a final volume of <500 μl. AMC008 SOSIP.v4.2-Avi was biotinylated to allow conjugation for FACS analysis. BirA biotin protein ligase (Avidity) was used for the biotinylation.

### Antibody production and purification.

HEK293F cells (catalog no. R79009; Invitrogen) were transfected to produce MAbs as described previously (16). In short, 62.5 μg of each heavy and light chain Ab DNA were cotransfected transiently in 250 ml HEK293F cells. Cell supernatant was harvested at 5 days by spinning down for 20 min at 4,000 × *g* and subsequent vacuum filtration with a 0.22-μm Steritop filter. Abs were purified from the culture supernatant by gravity-driven chromatography on a Protein G affinity column (Thermo Fisher Scientific). After addition of the supernatant, columns were washed twice with PBS before elution with 9 ml 0.1 M glycine (pH 2.0). Eluted Abs were collected in 1 ml 1 M Tris-HCl (pH 7.8). After elution, Abs were concentrated, by the use of 100-kDa Vivaspin filters (GE Healthcare) filters, to a final volume of <200 μl.

We named the isolated MAbs following McCoy et al. (16). In this system, each MAb is numbered according to their rabbit ID, followed by a unique alphabetical lineage identifier (A, B, C, etc.). Distinct lineage members received an additional number (A1, A2, A3, etc.).

### Single B-cell sorting.

Single B cells were selected and sorted as previously described (16). Briefly, biotinylated AMC008 SOSIP.v4.2-Avi Env proteins were conjugated to streptavidin (Strep)-allophycocyanin (APC) and Strep-fluorescein isothiocyanate (FITC) (both Thermo Fisher Scientific). Mouse-anti-rabbit IgG Ab conjugated to phycoerythrin (PE)-stained IgG on memory B cells (Southern Biotech). Conjugated Env protein was mixed with PBMCs to allow binding. We first selected IgG-positive (IgG^+^) cells and, within this population, sorted single B cells, positive for both APC- and FITC-conjugated AMC008.v4.2 Env trimer, into a lysis buffer consisting of RNase inhibitor (20 U; Thermo Fisher Scientific), 5× first-strand SuperScript III buffer (Invitrogen), 0.1 M dithiothreitol (DTT; Invitrogen), and Milli-Q water [MQ]. Sorted cells were immediately frozen at −80°C.

### Single-cell Ab RT-PCR, variable region gene amplification, and cloning.

A 6-μl aliquot of reverse transcription-PCR (RT-PCR) mixture (200 ng random hex primers [Thermo Fisher Scientific], 2 mM each dNTP mix [New England Biolabs], 50 U SuperScript III RTase, and MQ) was added directly to the sorted single B cells. The following PCR program was used to convert RNA into cDNA: 42°C for 10 min, 25°C for 10 min, 50°C for 60 min, and 95°C for 5 min. RT-PCR plates were used directly for variable region gene amplification using two subsequent PCRs. For both PCRs, 13 μl of PCR mix (MQ, 10× PCR buffer, dNTPs [10 mM], 0.25 U HotStar Plus polymerase [Qiagen], forward primer [25 mM], and reverse primer [25 mM]) was added subsequently to the RT-PCR plate and the PCR 1 plate. For PCR 1, 2 μl of RT-PCR product was added to this mix. PCR 1 was run at 95°C for 5 min; followed by 50 cycles of 94°C for 30 s, 58°C for 30 sec, and 72°C for 1 min; followed by 72°C for 10 min. For the heavy chain amplification, the annealing temperature was adapted to 48°C. For PCR 2, 2 μl of PCR 1 product was added to the PCR mix, and DNA was amplified at 95°C for 5 min; followed by 50 cycles of 94°C for 30 sec, 55°C for 30 sec, and 72°C for 1 min; followed by 72°C for 10 min. A final PCR 3 reaction using 1 μl of PCR 2 product was performed in MQ, 5× Phusion PCR buffer, dNTPs (10 mM), forward primers (25 mM), reverse primers (25 mM), and 0.2 U Phusion high-fidelity polymerase (New England Biolabs). The following PCR program was run: 98°C for 30 s; followed by 35 cycles of 98°C for 5 s, 68°C for 15 s, and 72°C for 20 s; followed by 72°C for 5 min.

Heavy and light chain-amplified variable regions were cloned by Gibson cloning into vectors expressing, respectively, the heavy or light chain constant region of rabbit Abs =. In short, 1 μl vector (45 ng) was incubated with 1 μl PCR 3 product and 2× Gibson mix (0.2 U T5 exonuclease [Epibio], 12.5 U Phusion polymerase [New England Biolabs], 2,000 U *Taq* DNA ligase [New England Biolabs], and Gibson reaction buffer [0.5 g PEG-8000 (Sigma Life Sciences), 1 M Tris/HCl (pH 7.5), 1 M MgCl_2_, 1 M DTT, 100 mM dNTPs, 50 mM NAD (New England Biolabs), and MQ]) for 60 min at 50°C.

### Mutant virus construction and production.

The infectious molecular clone (IMC) encoding replication-competent virus with AMC008 Env has been described previously (10). First, the AMC008 *env* fragment was transferred to pUC18 by traditional cloning methods using restriction enzymes SalI and BamHI (New England Biolabs). Mutations were then generated using the Q5 mutagenesis kit (New England Biolabs). Mutated AMC008 *env* fragments were cloned back into the original IMC by Gibson reactions. To produce virus stocks, HEK293T cells (CRL-11268; ATCC) were transfected with the IMCs using Lipofectamine 2000 (Invitrogen) and supernatants containing viruses were harvested 3 days later. Supernatants were directly frozen at −80°C.

### Neutralization assays.

Neutralization assays were executed as described previously (8, 15). In short, 1 in 3 dilution series were made of the various MAbs, starting at end concentrations ranging from 50 μg/ml to 100 μg/ml. Virus of interest was added to the diluted MAb and incubated for 1 h at room temperature. After incubation, the mixture was added to TZM-bl reporter cells (obtained through the NIH AIDS Reagent Program, Division of AIDS, NIAID, NIH, from John C. Kappes and Xiaoyun Wu) and incubated for 3 days at 37°C. IC_50_ values were determined as the concentration at which infectivity was inhibited by 50%.

For the decay neutralization assays, virus and MAb mixtures were incubated for 1, 3, 6, and 24 h at 37°C before addition to the TZM-bl reporter cells.

### Negative-stain electron microscopy.

Complex formation was performed by incubating AMC008 SOSIP.v4.2 trimers with a 6-fold molar excess of Fab for 3 h at room temperature. Complexes were subsequently diluted to 0.03 mg/ml in 1× Tris-buffered saline (TBS; pH 7.4) to achieve optimal particle density. Copper mesh grids were plasma cleaned for 20 s using a mix of argon and oxygen gas, and samples were stained with 2% uranyl formate for 50 s. For each data set, a FEI Tecnai Spirit (120 keV) with a Tietz (4k × 4k) camera was used in conjunction with the automated data collection software package Leginon (53). Data collection parameters included a magnification of ×56,000, a defocus of −1.5 μm, a pixel size of 2.05 Å per pixel, and a dose of 25 e^−^/Å^2^. Resulting images were stored in Appion (54); particles were picked with DoGPicker (55), stacked with a box size of 192 pixels, and processed using RELION (56). UCSF Chimera (57) was used for map segmentation and map/model docking.

### Binding and competition ELISAs.

Binding ELISAs were performed as previously described (2, 4). In short, ELISA plates were coated overnight with 100 μg Galanthus nivalis lectin (GNL) in 0.1 M NaHCO_3_ (pH 8.6) and blocked using casein (Thermo Fisher Scientific) at room temperature for 1 h after washing off the GNL with Tris-buffered saline (TBS). Env trimers were added at 2 μg/ml and incubated at room temperature for 2 h. Subsequently, Ab 3-fold serial dilutions were added for 2 h at room temperature at starting concentrations ranging from 1 μg/ml to 10 μg/ml. After washing with TBS, a 1:3,000 dilution of goat-anti-human or goat-anti-rabbit horseradish peroxidase (HRP, 1 μg/ml; SeraCare)-conjugated Ab was added and incubated at room temperature for 1 h. After washing with TBS plus 0.05% Tween 20, ELISA plates were developed using 1% 3,3′,5,5′-tetranethylbenzidine (Sigma-Aldrich), 0.01% H_2_O_2_, 100 mM sodium acetate, and 100 mM citric acid, and reactions were stopped after 3 min using 0.8 M H_2_SO_4_.

For HEK293T supernatant ELISAs, the same protocol was followed; however, 50 μl of HEK293T supernatant containing unpurified MAb was added instead of purified Abs.

For competition ELISA, His-tagged Env trimers were added at 2 μg/ml to precoated Ni-nitrilotriacetic acid (NTA) plates (Qiagen) and left at room temperature for 2 h. Blocking was achieved in 2% milk in TBS at room temperature for 1 h. The primary Ab (competitor) was added at excess (10 μg/ml) in 50 μl and incubated for 30 min at room temperature before the analyte Ab was added at a previously determined 70% effective concentration (EC_70_) in 50 μl and left at room temperature for another 1.5 h. Statistical significance was determined using a one-way analysis of variance (ANOVA) multiple-comparison test (Prism) comparing the sample without competitor to the corresponding sample with competitor present.

### Octet K2 biolayer interferometry kinetics and binding experiments.

All assays were conducted on the Octet K2 system (Bioforte). His-tagged AMC008 SOSIP.v4.2-His Env trimer (6 μg/ml) was captured on Ni-NTA sensors (Bioforte) for 600 s after baseline determination in PBS with 0.01% bovine serum albumin (BSA) and 0.002% Tween for 60 s. Association of a serial dilution of rabbit MAbs starting at 15 μg/ml was measured for 600 s by dipping the AMC008 SOSIP.v4.2 trimer-loaded sensor into a well containing MAb. Subsequently dissociation was measured in buffer (PBS, 0.01% BSA, and 0.002% Tween) for 600 s. Binding kinetics (*K_D_*, *K_a_*, and *K_d_*) were determined using a 1:1 fit model with independent fitting of *R*_max_ (Octet Data analysis software; Bioforte). Regeneration of the sensors was achieved through alternating cycles of 5 s in low-pH glycine buffer and a neutralization buffer (PBS, 0.01% BSA, and 0.002% Tween). After regeneration, sensors were used to recapture Env protein.

For competition analysis, after association of the competitor MAb to the SOSIP trimer bound to Ni-NTA sensors, a second 600-s association step was incorporated for the analyte MAb. Percent residual binding was calculated as follows: (shift in nm at 600 s of analyte binding × 100)/shift in nm of analyte in the absence of competitor binding.

### Surface plasmon resonance.

Surface plasmon resonance (SPR) experiments were done for kinetic and competition analysis of rabbit MAbs, as previously described (34). All assays were conducted on a Biacore 3000 instrument at 25°C. In all assays, HBS-EP (10 mM HEPES [pH 7.4], 150 mM NaCl, 3 mM EDTA, and 0.002% P20 surfactant) was used as running buffer (GE Healthcare). Briefly, anti-His Ab was covalently immobilized (15,000 response units [RU]) in all flow cells of a CM5 sensor chip by standard amine coupling. AMC008 SOSIP.v4.2-His Env trimer was captured on the anti-His-CM5 surface for both kinetic and competition analysis.

Competition assays were carried out with MAbs binding to the CD4bs (VRC01), gp120 V3-glycan (PGT121), and the gp120-gp140 interface (PGT151, 35O22, and ACS202). In the competition assays, MAbs were sequentially injected in the same cycle. The first Ab was injected for 200 s, immediately followed by the second Ab, at a flow rate of 30 μl/min. Dissociation followed for 300 s during the second injection. In addition, the second MAb was injected alone in a separate cycle.

The trimer immobilization levels and MAb concentrations were optimized previously to yield maximum self-competition. Rabbit MAbs (05A1, 05A2, and 05A3), PGT121, PGT151, and ASC202 showed 5 to 15% residual binding at a concentration of 1 μM and a trimer density of 500 RU, whereas VRC01 and 35O22 self-competed (15 to 20% residual binding) at a concentration of 1.5 μM and a density of ∼250 RU of AMC008 SOSIP.v4.2 trimer. Those MAb concentrations were used thereafter in the cross-competitions; for rabbit MAbs and VRC01 or 35O22, the trimer was captured to a level of 250 RU, whereas the other Abs (PGT121, PGT151, and ACS202) were done at a trimer capture level of 500 RU. The residual binding was calculated as follows: [(response difference at 200 s for the second Ab)/(response difference at 200 s for the same, second Ab when injected as a single Ab in a separate cycle)] × 100 (%). Significance was determined by a one-way ANOVA multiple-comparison test (Prism) comparing the sample without competitor to the corresponding sample with competitor present.

### Full-length SOSIP binding experiment.

Surface-expressed full-length AMC008.v4.2 SOSIP Env trimers were obtained by cotransfection of 10 μg gp160 SOSIP expression plasmid (39) and 2.5 μg furin expression plasmid into 1.75 × 10^6^ cells/ml HEK293F cells, as described above. At 60 to 65 h posttransfection, cells were spun down at 4,000 × *g*. 293F cells (1 × 10^5^) were added to each well of serially diluted MAbs and incubated for 2 h on ice. Cells were washed twice with PBS and subsequently stained in a volume of 20 μl containing 1:70 Alexa 647 (2 mg/ml)-conjugated mouse anti-human IgG (for 2G12 and PGT145 controls) (Invitrogen) or 1:70 PE (0.1 mg/ml)-conjugated mouse anti-rabbit IgG (Southern Biotech). Cells were stained for 45 min on ice, covered in foil, and subsequently washed with PBS and resuspended in 100 μl PBS and analyzed using a FACSCanto II analyzer (BD). Maximum mean fluorescent intensity (MFI) was calculated and plotted for each of the samples.

### Data availability.

Antibody sequences are available on request to the corresponding author. Variable domain sequences of heavy and light chains were uploaded in GenBank under accession numbers MZ170096, MZ170095, MZ170094, and MZ170097 for NAbs 05A1, 05A2, 05A3, and 07A1, respectively.
